# Actein Inhibits the Proliferation and Adhesion of Human Breast Cancer Cells and Suppresses Migration *in vivo*

**DOI:** 10.3389/fphar.2018.01466

**Published:** 2018-12-12

**Authors:** Xiao-Xiao Wu, Grace Gar-Lee Yue, Jin-Run Dong, Christopher Wai-Kei Lam, Chun-Kwok Wong, Ming-Hua Qiu, Clara Bik-San Lau

**Affiliations:** ^1^Department of Chemical Pathology, The Chinese University of Hong Kong, Hong Kong, China; ^2^Institute of Chinese Medicine, The Chinese University of Hong Kong, Hong Kong, China; ^3^State Key Laboratory of Research on Bioactivities and Clinical Applications of Medicinal Plants, The Chinese University of Hong Kong, Hong Kong, China; ^4^State Key Laboratory of Phytochemistry and Plant Resources in West China, Kunming Institute of Botany, Chinese Academy of Sciences, Kunming, China; ^5^State Key Laboratory of Quality Research in Chinese Medicines, Macau Institute for Applied Research in Medicine and Health, Macau University of Science and Technology, Taipa, China; ^6^Li Dak Sum Yip Yio Chin R & D Centre for Chinese Medicine, The Chinese University of Hong Kong, Hong Kong, China

**Keywords:** actein, cycloartane triterpenoid, metastasis, triple negative breast cancer, zebrafish embryos

## Abstract

**Background and purpose:** Metastasis is an important cause of death in breast cancer patients. Anti-metastatic agents are urgently needed since standard chemotherapeutics cannot diminish the metastatic rate. Actein, a cycloartane triterpenoid, has been demonstrated to exhibit anti-angiogenic and anti-cancer activities. Its anti-metastatic activity and underlying mechanisms were evaluated in the present study.

**Methods:** The effects of actein on the proliferation, cell cycle phase distribution, migration, motility and adhesion were evaluated using two human breast cancer cell lines, MDA-MB-231 (estrogen receptor-negative) and MCF-7 cells (estrogen receptor-positive) *in vitro*. Western blots and real-time PCR were employed to examine the protein and mRNA expression of relevant signaling pathways. A human metastatic breast cancer cell xenograft model was established in transparent zebrafish embryos to examine the anti-migration effect of actein *in vivo*.

**Results:** *In vitro* results showed that actein treatment significantly decreased cell proliferation, migration and motility. Furthermore, actein significantly caused G1 phase cell cycle arrest and suppressed the protein expression of matrix metalloproteinases of MDA-MB-231 cells. In addition, actein inhibited breast cancer cell adhesion to collagen, also reduced the expression of integrins. Actein treatment down-regulated the protein expression of epidermal growth factor receptor (EGFR), AKT and NF-κB signaling proteins. *In vivo* results demonstrated that actein (60 μM) significantly decreased the number of zebrafish embryos with migrated cells by 74% and reduced the number of migrated cells in embryos.

**Conclusion:** Actein exhibited anti-proliferative, anti-adhesion and anti-migration activities, with the underlying mechanisms involved the EGFR/AKT and NF-kappaB signalings. These findings shed light for the development of actein as novel anti-migration natural compound for advanced breast cancer.

## Introduction

Breast cancer is currently the second most lethal cancer in women, with continuous increase in incidence in recent decades (Torre et al., 2017). More than 90% of death caused by breast cancers are due to metastasis, a multistep process by which cancer cells disseminate from the primary tumor to localize at distant sites (Steeg, 2006). Despite the conventional therapeutic management of breast cancer, such as surgery, chemotherapy, radiation and hormonal therapy, these treatments are still ineffective in the control of breast cancer metastases or preventing relapses (Peart, 2015). Therefore novel anti-metastatic agents for breast cancer are urgently needed.

Actein is a natural triterpene glycoside compound isolated from the root of *Cimicifuga* species including *Cimicifuga racemosa*, *C. dahurica*, *C. foetida* as well as *C. heracleifolia*. *C. racemosa* is a well-known dietary supplement for women’s health in alleviating menstrual pain as well as for menopausal disorders to reduce the frequency and intensity of hot flashes in Europe (McKenna et al., 2001). In Asia, *C. dahurica*, *C. foetida*, and *C. heracleifolia* were reported to possess anti-osteoporosis, anti-viral, anti-diabetic, anti-malarial and vasoactive properties (Li and Yu, 2006). Previous *in vitro* studies have demonstrated that actein could inhibit the growth of breast cancer cells by synergizing with chemotherapy agents at previously suboptimal dosage (Einbond et al., 2006), induce calcium release, and modulate the nuclear factor-κB and Ras/Raf/mitogen-activated protein kinase/extracellular signal–regulated kinase pathways (Einbond et al., 2013). Our previous study showed that actein exhibited anti-angiogenic and anti-metastatic activities in mouse 4T1 mammary breast tumor-bearing model (Yue et al., 2016b). However, the potential influence of actein on anti-metastasis in human breast cancer has not been explored. The main objective of this study was to elucidate the *in vitro* and *in vivo* effects of actein on human breast cancer growth and initiation of metastasis and its underlying intracellular mechanisms. The proliferation, migration, adhesion and invasion of human estrogen receptor (ER)-negative breast cancer MDA-MB-231 cells and ER-positive MCF-7 cells were assessed upon exposure to actein. The further underlying mechanisms were performed on MDA-MB-231 cells because ER-negative breast cancer cells are more prone to metastasis than ER-positive cells (Bardou et al., 2003; Knutson and Lange, 2014). Cell cycle progression, extracellular matrix (ECM)-associated proteases, cell surface protein involved in AKT/NF-Kb signaling were determined upon actein treatment in MDA-MB-231 breast cancer cells. Another compound deoxyactein (DA), from *Cimicifuga* with similar structure of actein was used as control compound. Previous studies suggested that the growth inhibitory activity of *Cimicifuga* extracts appears to be related to their triterpene glycoside composition which is different between actein and DA (Einbond et al., 2008b). DA only exerts very minor effect on MCF-7 cell growth that could be ignored when compared to the potent effect of actein (Einbond et al., 2004). It was regarded as an inactive analog compound and therefore included in the *in vitro* cytotoxicity tests on MDA-MB-231 cells for comparison with actein.

Zebrafish (*Danio rerio*) is a well-developed model that has become a useful animal model for experimental research of cancer, immunology and stem cells (Anderson et al., 1974; Goessling et al., 2007; Stoletov and Klemke, 2008). The zebrafish embryo xenograft model has been established for the study of several human cancers including breast, prostate and colon cancers (Wagner et al., 2010; Drabsch et al., 2013; Francescangeli et al., 2015). Several aspects of tumor malignancies have been illustrated in embryos and adult zebrafish, such as cancer cell migration, proliferation, angiogenesis, extravasation, and inflammation (Mathias et al., 2007; Stoletov et al., 2007; Ellett and Lieschke, 2010; Feng et al., 2010). In addition, zebrafish embryos are suitable for drug discovery as they show high permeability for small-molecule chemical compounds (Peterson et al., 2004). Given that the adaptive immune system does not reach maturity until 4 weeks post-fertilization, cell graft-host rejection can be circumvented at early developmental stages (Casari et al., 2014). Therefore, 48 h post-fertilization (hpf) embryos were used in our study. Metastasis is a long and complex, multistep process requiring the establishment of primary tumor with subsequent dissemination, followed by colonization at a distant site. In human breast cancer xenograft zebrafish embryos model, MDA-MB-231 breast cancer cells can migrate from the yolk sac to the tail and head region, as well as major organs, such as heart and liver (Jung et al., 2012) which mimic metastatic colonization and outgrowth in a short time period (Chen et al., 2017). The transparency of embryo and larva allows the detection of fluorescent cell migration in the zebrafish at single cell level under fluorescent microscope.

## Materials and Methods

### Test Compounds, Chemicals and Reagents

**FIGURE 1:**
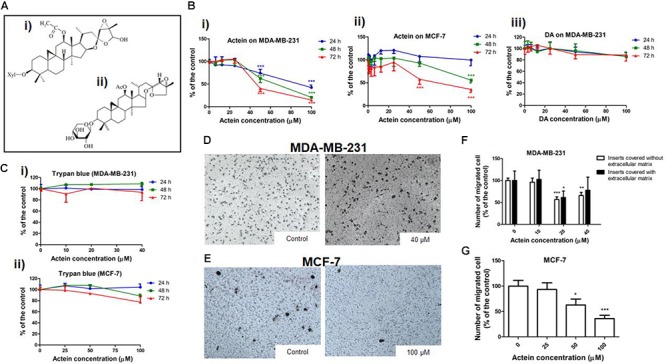
Actein inhibited cell migration in MDA-MB-231 and MCF-7 cells. **(A)** Chemical structure of **(i)** actein and **(ii)** DA. **(B)** Cytotoxic effects of actein (6.25 – 100 μM) on **(i)** MDA-MB-231 and **(ii)** MCF-7 cells, and **(iii)** DA on MDA-MB-231 cells upon 24, 48, or 72 h treatment were performed using MTT assay. Data were expressed as the mean fold of untreated controls (mean ± SD of 3 independent experiments with 5 replicates each). **(C)** Trypan blue assay of actein on **(i)** MDA-MB-231 cells and **(ii)** MCF-7 cells (mean ± SD of 3 independent experiments with 5 wells each). Cells were treated with actein for 5 h in transwell migration assay and invasion assay using matrigel. Representative photographs show the stained migrated **(D)** MDA-MB-231 cells or **(E)** MCF-7 cells on the lower side of the membrane after incubation. **(F,G)** Quantitative analysis summarized the number of migrated cells on the lower chambers (mean + SD of 3 independent experiments with duplicates each) and expressed as the percentage of the control. Differences among the treated and vehicle treated control groups were determined by one-way ANOVA. ^∗^*p* < 0.05, ^∗∗^*p* < 0.01, and ^∗∗∗^*p* < 0.001 as compared to control group.

Actein (Figure 1Ai) and DA (Figure 1Aii) were extracted and isolated from the root of *C. foetida* as previously described (Sun et al., 2011; Yue et al., 2016b). The rhizomes of C. foetida were collected in 2014 from Daju County, Lijiang Prefecture, Yunnan Province and identified by Prof. Pei Sheng-Ji, Kunming Institute of Botany, Chinese Academy of Sciences. A voucher specimen (KUN No. 20100906) has been deposited in the State Key Laboratory of Phytochemistry and Plant Resources in West China, Kunming Institute of Botany, Chinese Academy of Sciences, China. Actein and DA in dry powder form were dissolved in dimethylsulfoxide (DMSO) at a concentration of 100 mM as stock solutions, which were stored at -20°C and reconstituted in appropriate media prior to the experiments. DMSO (0.5% v/v) was used as the vehicle control.

The human breast cancer cell lines MDA-MB-231 (ER-negative) and MCF-7 (ER-positive) were purchased from American Type Culture Collection (ATCC, Manassas, VA, United States). RPMI 1640 medium, fetal bovine serum (FBS), penicillin-streptomycin, trypsin-EDTA, phosphate buffered saline (PBS), CM-Dil, anti-Rabbit IgG and HRP-Goat anti-Mouse IgG were obtained from Life Technologies (Life Technologies, Grand Island, NY, United States). MTT, propidium iodide (PI), tricaine, β-actin and RNase were obtained from Sigma-Aldrich (Sigma, St. Louis, MO, United States). Antibodies of MMP2, MMP9, tissue inhibitors of metalloproteinase (TIMP)-1 were purchased from Abcam (Cambridge, MA, United States) and CDK4, p21, Cyclin D1, phosphorylated Rb, epidermal growth factor receptor (EGFR), phospho (p)-EGFR, TIMP-2, AKT, pAKT, NF-κB, pNF-κB, IκBα and pIκBα were obtained from Cell Signaling Technology (Danvers, MA, United States). The fluorochrome (FITC or PE)-conjugated monoclonal antibodies, CD47b (integrin α2) and CD29 (integrin β1) were purchased from Miltenyi Biotec (Bergisch Gladbach, Germany). (Methyl-^3^H)-thymidine was obtained from PerkinElmer (Waltham, MA, United States). ECM cell adhesion array kit was purchased from Merck Millipore (Darmstadt, Germany). Real-time PCR reagent iTaq Fast SYBR Green Supermix was from Bio-Rad (Hercules, CA, United States). Transwell polycarbonate cell culture inserts (6.5 mm diameter, 8 mm pore size) were from Corning (Lowell, MA, United States).

### Cell Culture

Human MDA-MB-231 and MCF-7 cells were maintained in RPMI 1640 medium supplemented with 10% (v/v) heat-inactivated FBS and 1% (v/v) penicillin-streptomycin (PS). The cells were incubated in a humidified atmosphere with 5% CO_2_ at 37°C.

### MTT Cytotoxicity and Cell Proliferation Assay

The effects of cytotoxicity and proliferation of actein on MDA-MB-231 and MCF-7 cells were assessed using MTT assay, Trypan blue and (methyl-^3^H)-thymidine incorporation assay. Cytotoxicity effect of DA was also examined on MDA-MB-231 cells. Cells (5 × 10^4^/ml) were seeded in 96-well flat-bottom culture plate in 100 μl of medium and incubated at 37°C overnight. Actein in 100 μl of fresh medium was added to reconstitute the final concentrations (6.25–100 μM). Medium containing vehicle solvent DMSO [0.2% (v/v)] was added as untreated controls (Yue et al., 2016a). The effects of actein on the viable cell number were also determined by trypan blue assay as described in our previous study (Yue et al., 2016b).

### Transwell Migration Assay and Invasion Assay

The migration ability of cells was determined using a modified Boyden chamber in transwell migration assay (Leung et al., 2015) and invasion assay using matrigel (Debbie and Brooks, 2001). In the transwell migration assay, MDA-MB-231 and MCF-7 cells (5 × 10^4^/ml) in 100 μl of medium with 1% (v/v) FBS and actein in 100 μl medium [1% (v/v) FBS] were added to the upper chamber of each transwell inserts at final concentrations of 10 – 40 μM for MDA-MB-231 cells and 25 – 100 μM for MCF-7 cells in duplicate. Meanwhile, 500 μl of medium with 10% (v/v) FBS was added to the lower chamber. After incubation for 5 h at 37°C, cells were fixed with methanol for 3 min and stained with hematoxylin for 5 min. For the invasion assay using matrigel, the transwell inserts were covered with 20 μl basement membrane matrigel (1:8 dilution) first and the subsequent procedures were the same as transwell migration assay. They were photographed and the migrated cells were quantified by counting.

### Scratch Wound Assay

MDA-MB-231 and MCF-7 cells (2 × 10^5^/ml) were seeded onto 24-well plates overnight. The cells were starved with medium with 1% (v/v) FBS overnight and incubated with 10 μg/ml mitomycin C (Sigma-Aldrich) for 2 h prior to the scratch assay. According to the previously described procedure (Leung et al., 2015), two artificial crosses were created in the middle of wells by a pipette tip. Crosses of each well were photographed under Olympus IX71 microscope at 0 h and after incubation with different concentrations of actein. The percentages (%) of open wound area at final hour (22 h for MDA-MB-231 cells; 24 h for MCF-7 cells) against 0 h were calculated using the Tscratch software (CSELab, Zurich, Switzerland, Gebäck et al., 2009). The changes of open wound area represent the motility of cells across the scratch wound.

### Cell Surface Protein Analysis

Protein expression on MDA-MB-231 cell surface was studied by flow cytometry. Briefly, 5 × 10^5^ MDA-MB-231 cells in 7 ml culture medium were seeded in 100 mm culture dish and incubated overnight and then treated with actein (10 – 40 μM) for 24 h. After incubation, the attached and floating cells were harvested and washed twice with PBS. Cells were stained with fluorochrome (FITC or Phycoerythrin)-conjugated monoclonal antibodies, CD47b (integrin α2) and CD29 (integrin β1). After incubation at 4°C for 30 min in the dark, cells were washed twice with PBS prior to analysis. The fluorescence of 10,000 events was analyzed by the flow cytometer (FACSC Canto II flow cytometer, BD Biosciences, CA, United States).

### Cell Cycle Analysis

MDA-MB-231 cells (3 × 10^5^) were seeded onto a 6-well plate with 3 ml medium and incubated overnight. After cell starvation with 1% (v/v) FBS in RPMI medium for 24 h, RPMI medium containing 10% (v/v) FBS with actein (10 – 40 μM) were added into the wells for 24 or 48 h. The attached cells were collected and washed with PBS containing 5% (v/v) FBS twice. Cell pellets were fixed with 1 ml of 70% cold ethanol and stored at 4°C overnight. After washing, cells were re-suspended in PBS containing RNase A (10 μg/ml) and propidium iodide (20 μg/ml) and incubated for 30 min in the dark at 37°C. Flow cytometry was performed (10,000 events) to analyze the percentage of cells in different phases of cell cycle (CantoII flow cytometer).

### Gelatin Zymography

The activities of MMP-2 and MMP-9 (gelatinases) in MDA-MB-231 cells were assessed by gelatin zymography. Cells (5 × 10^4^/ml) were seeded in a 24-well plate overnight. The cells were then treated with actein (10 – 40 μM) in 0.5 ml FBS-free culture medium and incubated for 48 h. Medium was collected and centrifuged at 112 × g for 10 min. Supernatants were subjected to gelatin zymography as previously described (Yue et al., 2015).

### Extracellular Matrix Cell (ECM) Adhesion Assay

Extracellular matrix cell adhesion array kit was used to evaluate the effect of actein on MDA-MB-231 cell-ECM adhesion. Each well of an eight-well strip was pre-coated with one of the seven different human ECM proteins (collagen I, collagen II, collagen IV, fibronectin, laminin, tenascin, or vitronectin) or BSA as negative control. The assay was carried out according to the procedures recommended by the manufacturer. Briefly, cells were added to pre-coated wells treated with or without actein (10–40 μM) for 2 h. After washing, bound cells were stained and dissolved in an extraction buffer. The plate was read at 450 nm by a microplate reader. The change in optical density was represented as fold of untreated control.

### Western Blot Analysis

For Western blot analysis, MDA-MB-231 cells (1 × 10^6^/well) were seeded in a 100 mm dish and incubated overnight to allow attachment. Fresh medium with actein (10 – 40 μM) were added to the cells. After incubation for 24 or 48 h, cells were harvested, washed with PBS twice and lysed in lysis buffer for 30 min. The lysates were subjected to electrophoresis with 10% SDS-polyacrylamide gel and then transferred to a polyvinylidene difluoride (PVDF) membrane (Immobilon, Millipore) for protein expression analysis according to our previously described protocol (Yue et al., 2011). The blots were incubated overnight with primary antibodies against human β-actin, matrix metalloproteinases (MMP)2, MMP9, tissue inhibitors of metalloproteinase (TIMP)-1, p21, CDK4, Cyclin D1, phosphorylated Rb, EGFR, phospho (p)-EGFR, TIMP-2, AKT, pAKT, NF-κB, pNF-κB, IκBα, and pIκBα. After incubation with the secondary antibodies HRP-Goat anti-rabbit IgG and HRP-Goat anti-mouse IgG for 1 h, detection was performed using a molecular imager, ChemiDoc XRS+ (Bio-Rad Laboratory, Hercules, CA, United States).

### Real Time-PCR Analysis

MDA-MB-231 (1 × 10^6^) cells were seeded in a 100 mm dish and incubated overnight to allow attachment. Fresh medium with actein were added into the dish and the cells were treated for 24 or 48 h. The cell pellets were then collected and suspended in 1 ml Trizol reagent, total RNA from MDA-MB-231 cells were extracted, quantified and subjected to reverse transcription as described previously (Yue et al., 2011). The primer sequences are listed in Table 1. The specific gene mRNA levels were normalized with the internal control GAPDH mRNA level and then expressed as fold change compared to the control group.

**Table 1:** Gene specific PCR primers.

Gene name	Forward primer	Reverse primer
CXCR4	GCCCTCCTGCTGACTATTCC	GGCAGGATAAGGCCAACCAT


### Fluorescent Cell Labeling for Zebrafish Embryos

MDA-MB-231 cells were washed with PBS twice and transferred to a 1.5 ml Eppendorf tube resuspended in PBS containing the cell member dye CM-Dil (4 ng/μl) according to a published procedure (Jung et al., 2012). Briefly, the mixture of cells and staining dye were incubated at 37°C for 4 min followed by 15 min at 4°C. Cells were subsequently washed with PBS twice to remove the unincorporated dye, and the stained cells were re-suspended in PBS at the final concentration of 3 × 10^7^ cells/ml.

### Implantation of MDA-MB-231 Cells in Zebrafish Embryos

Transgenic zebrafish line Tg (*fli1:EGFP*)y1 was obtained from Zebrafish International Resource Center, University of Oregon (Eugene, OR, United States) and maintained as previously described (He et al., 2010). Healthy embryos were collected at their 48 hpf stage for experiments. Prior to the microinjection of cancer cells, the selected zebrafish embryos were anesthetized with tricaine (0.04 mg/ml) for 5 min. Zebrafish embryos were subsequently transferred onto a 2% agarose gel and excessive water was removed. About 300 prepared Dil-labeled breast cancer cells were injected into yolk sac of zebrafish embryo with non-filamentous borosilicate glass capillary needles attached to the microinjector under the microscopic examination (Nikon SMZ1500 microscope, Nikon Corp., Tokyo, Japan). Injected embryos were then transferred to a 6-well plate (30 embryos/well), and incubated with 6 mL of aquarium water containing actein or DA (40 and 60 μM, respectively) for the following 5 days at 28.5°C. DMSO (0.2% v/v) served as vehicle control.

### Fluorescent Imaging

The human breast cancer MDA-MB-231 cell migration in the zebrafish embryos was monitored every other day with a fluorescent microscope (Olympus IX71, Tokyo, Japan). Each living zebrafish embryo was picked up and placed onto a 2% agarose gel. The entire zebrafish embryo body was monitored to detect cancer cell distribution. For fluorescent imaging, the green filter was selected for CM-Dil stained cancer cells as recommended by manufacturer and blue filter for embryos (*EGFP*) (Ignatius and Langenau, 2011). The two different sets of images from the head to tail region were collected separately from each zebrafish embryo with 4X magnification to provide an overview of cancer cells. Two sets of images were merged together by ImageJ software (National Institutes of Health, MD, United States).

### Quantitation of Migration Cells in Zebrafish Embryos

Disseminated cancer cells in each zebrafish embryos were automatically counted using the software of Fiji at NIH (Schindelin et al., 2012) as previously described (Teng et al., 2013). Briefly, the stained cells remained in the yolk sac (the injection site) were masked off and eliminated in the images, while cells spread to the rest of the body of the zebrafish embryos were quantified using Fiji at single cell level. The extent of dissemination was reflected as numbers of cancer cell migrated from the injection site in different groups and thus the anti-migration effect of actein and DA could be assessed.

### Statistical Analysis

Data were expressed as mean + SD (*in vitro*) or mean + SEM (*in vivo*). Statistical analysis and significance were analyzed by one-way ANOVA followed by Dunnett *post hoc* test using GraphPad PRISM software version 6.0 (GraphPad Software, CA, United States). In all comparisons, *p* < 0.05 was considered as statistically significant.

## Results

### Actein Inhibited Cell Migration in MDA-MB-231 and MCF-7 Cells

The cytotoxicity of actein (Figure 1Ai) on breast cancer cells was determined by MTT and trypan blue assay after 24, 48 or 72 h incubation. Actein (3.25 – 40 μM) did not exert any significant cytotoxicity in MDA-MB-231 cells (Figure 1Bi). Also, actein (3.25 – 100 μM) did not show any effect on MCF-7 cells growth after 24 h treatment, while high dosages showed significant cytotoxicity on MCF-7 cells after 48 and 72 h treatment (Figure 1Bii). MDA-MB-231cell were incubated with DA (Figure 1Aii) with the same dosage as actein (0 – 100 μM) at same time points (24, 48, and 72 h). Results showed that DA has no significant cytotoxicity effect on MDA-MB-231 cells (Figure 1Biii). Trypan blue assay also showed that actein at tested concentrations did not significantly affect the viability of MDA-MB-231 and MCF-7 cells (Figures 1Ci,ii). The subsequent cell assays were performed using actein (<40 μM) on MDA-MB-231 cells and actein (<100 μM) for MCF-7 cells so that the inhibitory activities observed were not due to cytotoxicity.

The effects of actein treatment on breast cancer cell migration were evaluated in modified Boyden chambers covered with or without matrigel. After 5 h of incubation, cells migrated from the upper chamber to the lower chamber through the membrane as the chemoattractant gradient formed by 10% (v/v) FBS-containing culture medium in lower chamber. In the presence of actein (20 and 40 μM), the number of migrated MDA-MB-231 cells significantly decreased, by 43% and 34%, respectively (Figures 1D,F). For inserts covered with Matrigel, the migrated MDA-MB-231 cells significantly decreased in 20 μM (Figure 1F). Moreover, actein at 50 and 100 μM significantly inhibited MCF-7 cells migration by 27 and 64% (Figures 1E,G), thereby suggesting the inhibitory effect of actein on the migration of human breast cancer cells.

### Actein Inhibited Cell Motility in MDA-MB-231 and MCF-7 Cells

**FIGURE 2:**
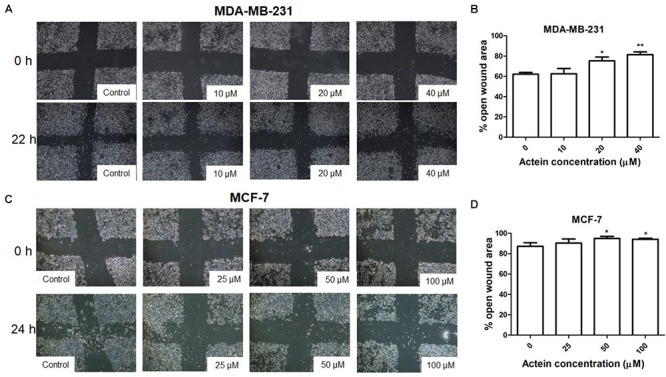
Effect of actein on cell motility of breast cancer cells. Representative photomicrographs showing the effect of actein on **(A)** MDA-MB-231 or **(C)** MCF-7 cells motility, determined by the open wound area after 22 or 24 h of incubation (*n* = 12). **(B,D)** Quantification of wound-induced cell motility is shown. Results are expressed as the mean open wound area (mean + SD of 4 independent experiments). Differences among the treated and vehicle treated control groups were determined by one-way ANOVA with. ^∗^*p* < 0.05 and ^∗∗^*p* < 0.01 as compared to control group.

To determine the efficacy of actein against cancer cell motility *in vitro*, the scratch wound healing assay were employed (Leung et al., 2015). Actein (20 and 40 μM) increased the remaining open wound area after 22 h of MDA-MB-231 cell migration (Figures 2A,B). Similarly, after 24 h incubation, actein (50–100 μM) also increase the open wound area in MCF-7 cells (Figures 2C,D), indicating that actein could inhibit the motility of breast cancer cells.

### Actein Inhibited Cell Proliferation and Induced Cell Arrest in G1 Phase

**FIGURE 3:**
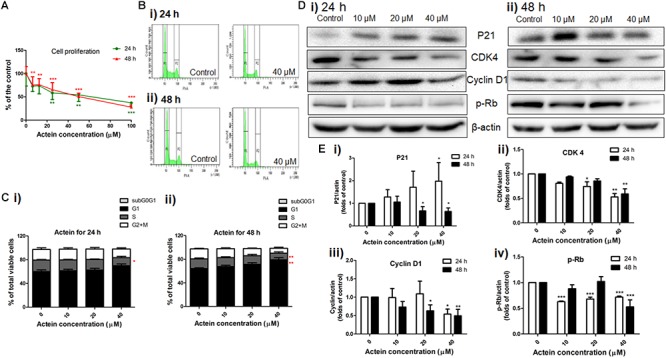
Actein inhibited cell proliferation and mediated cell arrest in G1 phase through the modulation of p21-cyclin D1/CDK4-pRb pathway. **(A)** Effect of actein on MDA-MB-231 cell proliferation was investigated by (methyl-^3^H)-thymidine incorporation assay after 24 or 48 h incubation with actein. Results are expressed as mean ± SD of 3 independent experiments. **(B)** Cell cycle of MDA-MB-231 cells treated with actein for **(i)** 24 and **(ii)** 48 h were evaluated by flow cytometry, and **(C)** Results were presented as mean + SD of 3 independent experiments in bar charts. **(D)** Western blot analysis of p21-cyclin D1/CDK4-pRb pathway related proteins in MDA-MB-231 cells upon **(i)** 24 or **(ii)** 48 h treatment with actein (10 – 40 μM). **(E)** Bar charts show the expression levels of proteins of **(i)** P21, **(ii)** CDK4, **(iii)** Cyclin D1, and **(iv)** phosphorylated Rb, which were adjusted with corresponding β-actin protein level and expressed as fold of control (mean fold of control + SD from 6 independent experiments). Differences among the treated and vehicle treated control groups were determined by one-way ANOVA. ^∗^*p* < 0.05, ^∗∗^*p* < 0.01, and ^∗∗∗^*p* < 0.001 as compared to control group.

As MDA-MB-231 cells were more sensitive to the inhibitory activities of actein than MCF-7 cells, MDA-MB-231 cells were chosen to evaluate the effect of actein in the subsequent experiments. The MDA-MB-231 cell proliferation was significantly inhibited by actein (6.25 – 100 μM) after 24 or 48 h treatment in a concentration-dependent manner (Figure 3A). Further investigation of the effect of actein on cell cycle distribution were analyzed by flow cytometry after treatment with actein for 24 or 48 h (Figures 3Bi,ii). Figure 3C demonstrated that actein significantly increased the percentage of cells in G1 phase with a corresponding decrease in the S phase. In order to further investigate the involvement of cyclins and cyclin-dependent kinases during G1 phase arrest, Western blot was performed to determine the expressions of CDK4, P21, Cyclin D1 and phosphorylated Rb (Figure 3D). As shown in Figure 3E, the expression of CDK4, Cyclin D1 and phosphorylated Rb were significantly inhibited after 24 and 48 h incubation with actein, while the expression of p21 was significantly increased at 24 h but inhibited at 48 h.

### Actein Reduced ECM Degradation in MDA-MB-231 Cells

**FIGURE 4:**
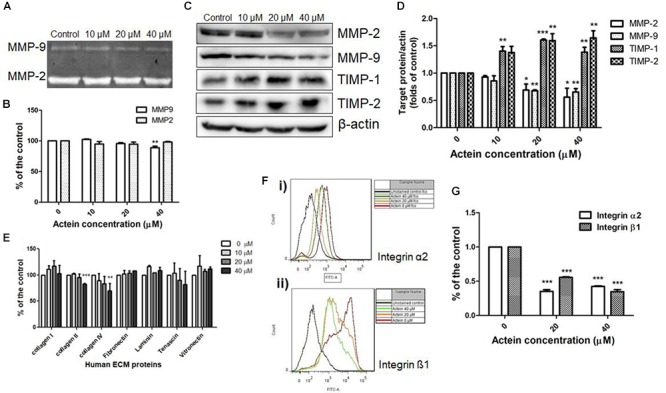
Effects of actein on cell adhesion to ECM proteins and matrix degradation. **(A)** Representative photographs show the activities of MMP-2 and MMP-9 on the stained gelatin gel. **(B)** The effects on MMP9 and MMP2 activities were represented by the digested gelatin on the gel in gelatin zymography assay. Results were expressed as fold of untreated controls (mean + SD of 4–5 independent experiments in duplicate). **(C)** Representative Western blots show the effect of actein on the expressions of MMP-2, MMP-9, TIMP-1, and TIMP-2 of MDA-MB-231 cells after 24 h treatment with or without actein. **(D)** The histograms show the quantified results of the expressions of the ECM-associated proteinases, which were normalized with corresponding β-actin protein expression and expressed as fold of untreated controls (mean + SD of 4 independent experiments). **(E,F)** Effects of actein on breast cancer cell adhesion. **(E)** In ECM-adhesion assay, MDA-MB-231 cells treated with or without actein were added to wells pre-coated with ECM proteins for 2 h. Results were expressed as the mean fold of the untreated controls (mean + SD of 3 independent experiments in duplicates). **(F)** Representative flow cytometric histograms show the inhibitory effect of actein on **(i)** integrin α2 and **(ii)** β1 expressions. **(G)** Bar charts show the expressions of integrins α2 and β1 using flow cytometry and expressed as fold of control (mean + SD of 4 independent experiments). Statistical differences were determined by One-way ANOVA, ^∗^*p* < 0.05, ^∗∗^*p* < 0.01, and ^∗∗∗^*p* < 0.001 against untreated control.

Matrix metalloproteinases (MMP)-2 and 9 are ECM-associated proteases involved in matrix degradation. Gelatin zymography assay revealed the activities of MMP-9 and MMP-2 secreted by MDA-MB-231 cells. As shown in Figures 4A,B, actein (40 μM) significantly inhibited MMP-9 activity, while no significant effect has been found on the activity of MMP-2. The effects of actein on the expression of ECM-associated proteinases, MMP-2, MMP-9 and tissue inhibitor TIMP-1, TIMP-2 in MDA-MB-231 cells were assessed using Western blot (Figure 4C). Actein (20 and 40 μM) could significantly reduce the expression of MMP-9 as well as MMP-2 in MDA-MB-231 cells (Figure 4D). TIMP-1 and TIMP-2 are MMP inhibitors for MMP-9 and MMP-2, respectively. We found that actein at 20 and 40 μM significantly enhanced the expression of TIMP-1 and TIMP-2 after 24 h incubation (Figure 4D). This indicates that actein can upregulate the expression of inhibitors TIMP-1 and TIMP-2 but downregulate the expression of MMP-9 and MMP-2, thereby resulting in the suppression of cancer metastasis.

### Actein Reduced Breast Cancer Cells Adhesion to ECM Proteins

The ability of cancer cells adhere to matrix proteins was assessed by ECM adhesion assay. Actein inhibited breast cancer cell adhesion to ECM proteins (Figure 4E).

### Actein Reduced the Expression of Integrins and Integrin Binding in Breast Cancer Cells

Integrins are involved in intercellular or cell-matrix adhesion. Results of flow cytometry revealed that actein (20 – 40 μM) significantly reduced the expression of integrin subunits α2 and β1 on MDA-MB-231 cells (Figures 4F,G). Since actein reduced matrix-cell adhesion and integrin expression of MDA-MB-231 cells, it is suggested that actein can reduce matrix-cell adhesion via reducing integrin binding.

### Actein Down-Regulated the Expression of Proteins Involved in EGFR, AKT and NF-κb Signaling

**FIGURE 5:**
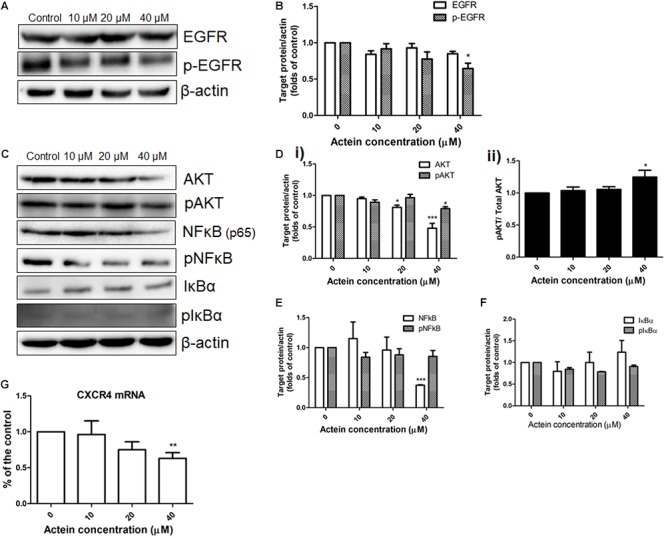
Effects of actein on expression of proteins in various signaling pathway in MDA-MB-231 cells. **(A,C)** Representative Western blots shows the expressions of EGFR signaling pathway related proteins in MDA-MB-231 cells. After MDA-MB-231 cells were treated with or without actein (10 – 40 μM) for 24 h, whole cell lysate was subjected to Western blotting. **(B,D,E,F)** Bar charts shows the results of proteins EGFR, p-EGFR, AKT, pAKT, NF-κB, pN-FκB, IκBα, and pIκBα using Western blot analysis, which were normalized with corresponding β-actin protein expression and expressed as fold of control (mean + SD of 4 independent experiments). **(G)** Quantitative RT-PCR analysis shows the mRNA expressions of CXCR4 in MDA-MB-231 cells after treatment with or without actein. Data were normalized to corresponding human GAPDH expressions. mRNA expressions results are expressed as fold of control. Statistical differences were determined by one-way ANOVA, with ^∗^*p* < 0.05, ^∗∗^*p* < 0.01, and ^∗∗∗^*p* < 0.001 against untreated control.

The effect of actein (10 – 40 μM) on EGFR and pEGFR of MDA-MB-231 cells was investigated using Western blot (Figure 5A). Actein (40 μM) significantly reduce the expression of phosphorylated EGFR after 24 h treatment, but without any significant effect on EGFR expression (Figure 5B).

AKT and NF-κB signaling pathways were also investigated in MDA-MB-231 cells with or without actein treatment for 24 h (Figure 5C). Results showed that actein (40 μM) significantly reduced the expressions of AKT and pAKT (Figure 5Di) while the ratio of pAKT/ Total AKT was increased (Figure 5Dii). To determine the effects of actein on NF-κB signaling transduction, the phosphorylation of both IκBα and NF-κB in the whole cell extracts of MDA-MB-231 cells were studied. Actein at 40 μM significantly reduced the NF-κB (p65) while the levels of phosphorylated NF-κB were unchanged in MDA-MB-231 cells (Figure 5E). Actein (20 – 40 μM) was found to upregulate the expression of IκBα at 24 h treatment (Figure 5F).

### Actein Reduced CXCR4 mRNA Expression in MDA-MB-231 Cells

The mRNA expression of CXCR4 in the MDA-MB-231 cells were determined by real-time PCR analysis. The mRNA expression of CXCR4 was downregulated by actein treatment (Figure 5G).

### Actein Suppressed Breast Cancer Cell Migration in Zebrafish Embryos

**FIGURE 6:**
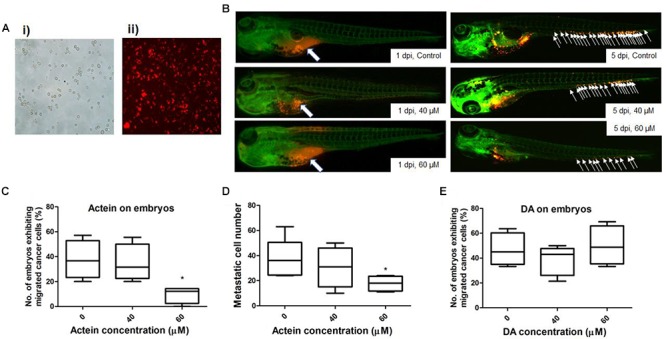
Actein decreased breast cancer cell migration in the zebrafish embryos. **(A)** Representative images of MDA-MB-231 cells before **(i)** and after **(ii)** CM-Dil staining (100 × magnification) are shown. **(B)** Representative images show breast cancer cells visualized by the red fluorescent dye (white arrows) (*n* = 360). The images on the left panel represent embryos at 1 day post injection with all cells remained in the yolk sac. White arrows in the images on the right panel indicate breast cancer cells disseminated from the injection site (yolk sac) at 5 dpi. **(C)** The number of embryos exhibiting migrated cancer cells was significantly lower in 60 μM treatment group compared to the control group. The results were represented as the mean + SEM of 4 independent experiments (*n* = 360). **(D)** Fiji software analysis on the images (mean + SEM of 4 independent experiments). **(E)** DA shows no effect on the embryos exhibiting migrated cancer cells. Statistical differences were determined by One-way ANOVA, ^∗^*p* < 0.05 vs. untreated control.

The number of zebrafish embryos exhibiting migrated cancer cells was defined as number of embryos with more than 5 cells outside the yolk sac region (Teng et al., 2013). MDA-MB-231 cells were stained with CM-Dil dye (Figure 6A). Injected cells remained in the injected site at 1 day post injection (dpi) (Figure 6B). At 5 dpi, MDA-MB-231 cells migrated outside the yolk sac. The number of embryos with migrated cells in actein (60 μM) treatment group significantly decreased by 74% when compared to the control group (Figure 6C). Moreover, the number of migrated cells in embryos varies among different groups at 5 dpi (Figure 6B). After quantification, results showed that the number of migration cells in actein (60 μM)-treated embryos also significantly decreased (Figure 6D), thereby showing the anti-proliferation and anti-migration effects of actein in the zebrafish xenograft model. Moreover, the survival rate of zebrafish embryos was not affected by actein treatments (Supplementary Figure S1) suggesting that the actein at testing doses did not cause observable toxicity to the zebrafish embryos. Moreover, the number of embryos with migrated cells after treatment with inactive control compound DA did not show any significant difference from that of the untreated control embryos (Figure 6E).

## Discussion

In the present study, actein, a natural triterpene glycoside compound isolated from the rhizome of *C. foetida* (Chinese herb “shengma”), was shown to inhibit proliferation and invasion of human ER negative breast cancer MDA-MB-231 cells *in vitro* and human breast cancer migration in the zebrafish xenograft model. Previous studies have showed that actein exhibited the anti-proliferative activities on human breast cancer cells (Einbond et al., 2004, 2008a) and osteoblastic cells (Lee and Choi, 2014), but with no further study on its anti-metastatic effect on advanced human ER negative breast cancer. According to our previous study, actein exhibited anti-angiogenic and anti-metastatic activities in a 4T1 mammary breast tumor-bearing model (Yue et al., 2016b). In the present study, we demonstrated for the *in vivo* anti-migration effect of actein by using the breast cancer cell xenotransplantation zebrafish embryos model.

Metastasis is a complex, multiple step and multifunctional cascade through which cancer cells spread from the primary tumor to distant anatomical sites (Spano et al., 2012). These cascades include several steps, such as angiogenesis and the subsequent escape of tumor cells from the primary tumor mass, and the invasion and migration through the basement membrane and ECM surrounding the tumor epithelium (Kozłowski et al., 2015). Our *in vitro* results have shown that actein could inhibit the growth of MDA-MB-231 and MCF-7 cells, while the related compound DA had no significant effect on MDA-MB-231 cells and remained as an inactive analog compound. MDA-MB-231 cells were used in the studies of underlying intracellular mechanisms of the anti-migration effect of actein since ER-negative breast cancer cells are more prone to metastasis than ER-positive cells.

Cancer cells have uncontrolled proliferation compared to the normal cells, thereby leading to severe metastasis at different distinct organs. Actein (6.25 μM) could down-regulate breast cancer cell proliferation which helps in attenuating the degree of metastasis as well as inhibition of primary tumor growth (Yue et al., 2016b). Actein suppressed the cell cycle transition from G1 to S phase (Figure 3C). The primary G1/S cell cycle checkpoint controls the commitment of cells to transit through the G1 phase to DNA synthesis S phase. Notably, two cell cycle kinase complexes, CDK4/6-Cyclin D and CDK2-Cyclin E, work together to relieve the inhibition of dynamic transcription complex that contains the retinoblastoma (Rb) protein and E2F (Hanai et al., 2002; Min et al., 2004). To further explore the underlying mechanism of actein in G1 phase, the down-regulation of phosphorylated Rb and the corresponding inhibition of relevant cell cycle protein cyclin D1 and CDK4 expressions were observed using Western blot analysis. As the CDK inhibitors, p21 was expected to have higher expression to inhibit cell proliferation and cell cycle G1 phase arrest (Harper et al., 1993; Toyoshima and Hunter, 1994; Lee et al., 1995). However, expression of p21 was significantly inhibited by actein treatment with 48 h which is in contrast to the previous study (Einbond et al., 2004). In fact, the expression of p21 in cells treated with 20–40 μM actein for 24 h was upregulated, which was consistent to the results from Einbond’s group. Prolonged exposure time to actein may further alter the expression of p21 in MDA-MB-231cells. Nevertheless, results suggested that actein inhibited DNA synthesis and cell cycle progression which may be due to the regulation of CDK4-Cyclin D activity.

The results from invasion assay using matrigel showed that actein could inhibit breast cancer cells passing through the ECM directly (Figure 1F). ECM not only acts as a barrier to invasion, but also involves in cell-ECM interactions during invasion. Our ECM adhesion assay data revealed that actein significantly inhibited cell adhesion of MDA-MB-231 to collagen II and collagen IV which are the major components of the ECM (Figure 4E). Actein also inhibited the expressions of integrin α2 and β1 which are responsible for cellular adhesion onto collagen. The inhibition of their expression likely contributed to the anti-adhesive activity of actein on ECM-cell adhesion. Apart from cell adhesion, adhesion proteins on cancer cells actually play multiple roles in metastasis, such as the regulation of cell migration, proliferation, invasion and angiogenesis (Jin and Varner, 2004). When migrating cancer cells adhere to ECM, degraded ECM provides a path for invasive cancer cells during metastasis. Therefore, inhibiting the expression and activity of ECM-associated proteinases are responsible for inhibiting matrix degradation and invasion. MMPs play important roles in the degradation of the ECM for the migration of breast cancer cells throughout the body (Köhrmann et al., 2009). Our results demonstrated that MMP-9 activities in MDA-MB-231 cells were reduced after actein treatment, suggesting that actein could inhibit breast cancer cell migration via reducing the degradation of ECM and hence cancer cell invasion. As TIMPs, a family of small extracellular proteins, play a role in inhibiting MMP activity, low levels of TIMPs in cancer cells are in favor of cancer progression, invasion and metastasis (Valente et al., 1998). Results for the expressions of TIMP1,2, upon 24 h actein treatment, demonstrated that actein significantly upregulated the expressions of TIMP1 and TIMP2, thereby resulting in reduced cancer progression and cancer cell invasion.

EGFR signaling pathway plays a key role in cancer cell survival, growth and metastasis (Zhang et al., 2007; Capdevila et al., 2009). EGFR is overexpressed in triple negative breast cancer. It is important to identify novel drugs that can mediate EGFR pathway to inhibit cancer growth and metastasis, especially in triple negative breast cancer patients. In our present study, actein suppressed the expression of phosphorylation of EGFR in human triple negative MDA-MB-231 cancer cells. AKT and NF-κB signaling pathways are usually found in highly invasive cancer cells such as MDA-MB-231 cells (Sweeney et al., 2004). It has been reported that EGFR activation can strongly stimulate AKT and NF-κB signaling pathway (Zhang et al., 2014). AKT is one of downstream targets of EGFR pathway and there is correlation between high levels of NF-kB activation and EGFR overexpression in breast cancer (Biswas and Iglehart, 2006; Seshacharyulu et al., 2012). Therefore, the effect of actein on AKT and NF-κB signaling pathway was also examined. Actein was shown to inhibit the expression of pAKT, AKT and NF-κB. However, the ratio of pAKT/AKT was increased and AKT phosphorylation has been shown to correlates with cell apoptosis inhibition (Itoh et al., 2002). Results illustrated that actein might exert its anti-metastatic activity through inhibiting EGFR, AKT and NF-κB signaling pathway, following activity of ECM-associated proteases including MMP-9. Previous reports suggested a crucial role of high levels of chemokine receptor CXCR4 expression in the metastatic process (Kang et al., 2003; Darash-Yahana et al., 2004; Smith et al., 2004). Severe lung metastases of breast cancer can induce secondary bone metastases, attract cancer cells and regulate proliferation and invasion at specific metastatic sites (Smith et al., 2004; Roato, 2014). Therefore, MDA-MB-231 cells treated with or without actein were subjected to real-time PCR to assess the regulation of CXCR4 gene expression on cancer cells. Our results indicated that actein could down-regulate the CXCR4 gene expression in breast cancer cells.

*In vivo* study adopted the zebrafish xenograft model by injecting malignant breast cancer cells into the embryos and monitoring their invasion and migration within the zebrafish body (Drabsch et al., 2013). Molecular and cellular components involved in tumorigenicity are highly conserved between zebrafish and human to provide a feasible animal platform for examining the effect of actein on human metastatic breast cancer. Several methods of transplantation can be applied in combination with different fluorescent zebrafish lines such as the yolk sac injection and eyes injection (Jung et al., 2012; Chen et al., 2015). In this study, injection of MDA-MB-231 cells into the yolk sac was applied to test for the anti-migration activity of actein on breast cancer. Similar to other animal models, there are pros and cons of using zebrafish xenograft model to study cancer cell migration. Apart from the main advantage of optical transparency of the embryos, the advantages of using zebrafish embryos model also include the ease of caring than rodents and shorter test duration with lower cost (Hill et al., 2005), which enables the zebrafish embryos transplantation model to be a useful screening platform for natural product bio-activity. Given the rapid turnaround time, such system can be utilized to probe the aggressive behavior of not only human cell lines but also freshly isolated samples from human cancer patients. However, it is obvious that the human cancer cells injected into these zebrafish embryos are not operating in a syngeneic milieu and some interplay with the stroma may consequently be lost (Berens et al., 2016). Despite these limitations, the zebrafish embryo model has a deserved place in both basic and translational cancer research. In our study, zebrafish embryo model plays important role in testing the anti-migration effect of actein within a short time period.

## Conclusion

In conclusion, our findings suggested that actein could inhibit breast cancer cells metastasis, which may act through the inhibition of EGFR/AKT and NF-κB signaling, suppressed cell adhesion to ECM proteins as well as the reduction of ECM degradation and CXCR4 expression. The breast cancer xenograft zebrafish model has demonstrated for the first time of the anti-migration effect of actein in zebrafish embryos without toxicity. The present findings therefore reveal that actein can be a potential anti-metastatic candidate drug for the treatment of advanced human breast cancer.

## Ethics Statement

This study was carried out in accordance with the guidelines of laboratory animal care and the experimental protocols have been approved by the Animal Experimentation Ethics Committee of the Chinese University of Hong Kong (Ref no. 16/162/MIS and 16/196/MIS).

## Author Contributions

X-XW, C-KW, GY, and CB-SL designed the research study. X-XW and GY performed the biological research and analyzed the data. J-RD and M-HQ performed the chemical research. C-KW and CB-SL contributed essential reagents and research facilities. X-XW, GY, CB-SL, and CW-KL wrote the manuscript. All authors reviewed the manuscript.

## Conflict of Interest Statement

The authors declare that the research was conducted in the absence of any commercial or financial relationships that could be construed as a potential conflict of interest.
