# Supplementary material for: Actein Inhibits the Proliferation and Adhesion of Human Breast Cancer Cells and Suppresses Migration in vivo
Source: Front Pharmacol. 2018 Dec 12;9:1466. doi: 10.3389/fphar.2018.01466 (PMC6299023; doi:10.3389/fphar.2018.01466)
Supplement: Supplementary file 1 [file Image_1.pdf]

Supplementary Figure

Actein inhibits the proliferation and adhesion of human breast cancer cells and suppresses migration *in vivo*

Xiao-Xiao Wu[1,#], Grace Gar-Lee Yue[2,3,#], Jin-Run Dong[4,#], Christopher Wai-Kei Lam[5], Chun-Kwok Wong[1,2,3,6,*], Ming-Hua Qiu[4,*], Clara Bik-San Lau[2,3,*]

[1]Department of Chemical Pathology; [2]Institute of Chinese Medicine; [3]State Key Laboratory of Phytochemistry and Plant Resources in West China, The Chinese University of Hong Kong, Shatin, New Territories, Hong Kong SAR, China; [4]State Key Laboratory of Phytochemistry and Plant Resources in West China, Kunming Institute of Botany, Chinese Academy of Sciences, Kunming, China; [5]State Key Laboratory of Quality Research in Chinese Medicines, Macau Institute for Applied Research in Medicine and Health, Macau University of Science and Technology, Taipa, Macau; [6]Li Dak Sum Yip Yio Chin R & D Centre for Chinese Medicine, The Chinese University of Hong Kong, Hong Kong SAR, China.

***Corresponding authors**

Clara Bik-San LAU, Institute of Chinese Medicine, The Chinese University of Hong Kong, Shatin, New Territories, Hong Kong. Tel: +852 3943 6109, Fax: +852 2603 5248, E-mail: claralau@cuhk.edu.hk

Ming-Hua QIU, State Key Laboratory of Phytochemistry and Plant Resources in West China, Kunming Institute of Botany, Chinese Academy of Sciences, China. Tel : +86 871 6522 3257, Fax : +86 871 6522 3255, E-mail : mhchiu@mail.kib.ac.cn

Chun-Kwok WONG, Department of Chemical Pathology, Prince of Wales Hospital, Shatin, New Territories, Hong Kong. Tel: +852 3505 2964, Fax: +852 2636 5090, E-mail: ck-wong@cuhk.edu.hk

# These authors contributed equally to this work.

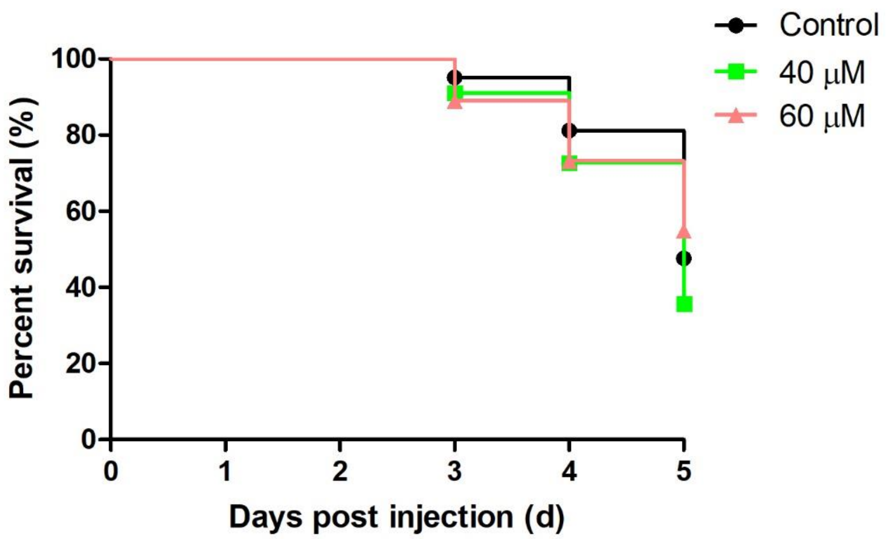

Figure S1: Survival rate of embryos after treated with or without actein for the following 5 days. Results were expressed as ratio of alive embryos per group in treated and vehicle-treated control of 4 independent experiments.
